# Computational/experimental evaluation of liver metastasis post hepatic injury: interactions with macrophages and transitional ECM

**DOI:** 10.1038/s41598-019-51249-y

**Published:** 2019-10-21

**Authors:** Shanice V. Hudson, Hunter A. Miller, Grace E. Mahlbacher, Douglas Saforo, Levi J. Beverly, Gavin E. Arteel, Hermann B. Frieboes

**Affiliations:** 1 Department of Pharmacology and Toxicology, University of Louisville, Louisville, KY 40292 USA; 2 Department of Bioengineering, University of Louisville, Louisville, KY 40292 USA; 3 Present Address: Department of Medicine, Division of Gastroenterology, Hepatology, and Nutrition, University of Pittsburgh, Pittsburgh, PA 15261 USA; 4 University of Louisville Alcohol Research Center, University of Louisville, Louisville, KY 40292 USA; 5 James Graham Brown Cancer Center, University of Louisville, Louisville, KY 40292 USA

**Keywords:** Cancer models, Biomedical engineering

## Abstract

The complex interactions between subclinical changes to hepatic extracellular matrix (ECM) in response to injury and tumor-associated macrophage microenvironmental cues facilitating metastatic cell seeding remain poorly understood. This study implements a combined computational modeling and experimental approach to evaluate tumor growth following hepatic injury, focusing on ECM remodeling and interactions with local macrophages. Experiments were performed to determine ECM density and macrophage-associated cytokine levels. Effects of ECM remodeling along with macrophage polarization on tumor growth were evaluated via computational modeling. For primary or metastatic cells in co-culture with macrophages, TNF-α levels were 5× higher with M1 vs. M2 macrophages. Metastatic cell co-culture exhibited 10× higher TNF-α induction than with primary tumor cells. Although TGFβ1 induction was similar between both co-cultures, levels were slightly higher with primary cells in the presence of M1. Simulated metastatic tumors exhibited decreased growth compared to primary tumors, due to high local M1-induced cytotoxicity, even in a highly vascularized microenvironment. Experimental analysis combined with computational modeling may provide insight into interactions between ECM remodeling, macrophage polarization, and liver tumor growth.

## Introduction

Causes of liver disease include alcohol or toxicant exposure, obesity, and viral infection, among a host of cofactors. Resulting changes to the extracellular matrix (ECM) appear to play key mechanistic roles in both oncogenic and non-oncogenic diseases^1^. These changes are generally mediated by modulation of the normal balance between *de novo* synthesis and deposition, ECM-degrading enzymes (e.g., matrix metalloproteinases; MMPs) and degradation inhibitors (e.g. tissue inhibitors of metalloproteinases, TIMPs). In cancer progression, the balance between these mediators of ECM homeostasis is nuanced; although cancers tend to induce MMP activity to facilitate tumor cell invasion, they often increase *de novo* ECM deposition. In liver fibrosis, ECM synthesis and deposition are increased, while TIMPs are upregulated and MMPs impaired, so that the balance tips towards aberrant matrix deposition. Further, ECM remodeling due to normal wound healing as well as liver diseases employs developmental pathways highly associated with cancer progression^2^, thus conferring invasive, migratory, and proliferative potential to metastatic cells that seed in this niche^3^.

Alcohol exposure has been specifically reported to induce transitional changes to the hepatic matrisome^4^. These changes contribute to liver inflammation and the pathological hallmarks of liver injury, including activation of innate immune responses^5^. Additionally, circulating monocytes and resident macrophages are recruited by tumors to facilitate neoangiogenesis and proliferation. In turn, macrophage signaling and ECM turnover both in cancer and in liver diseases involve macrophage modulation of the microenvironment^6^, partly mediated, by altered macrophage polarization. In the context of tumor associated macrophages (TAMs), although perivascular TAMs present with a proinflammatory (‘classical’ M1) phenotype, stromal macrophages bordering hypoxic regions, exhibit a more anti-inflammatory (‘alternative’ M2) trophic phenotype^7^. These M2-like TAMs release pro-angiogenic factors such as vascular endothelial growth factor (VEGF) and angiopoietin 2 (Ang2) in response to hypoxic cell signaling^8,9^. It remains poorly understood, however, to what extent the interactions of pro- and anti-inflammatory macrophage and ECM remodeling in the context of liver disease affect metastatic tumor growth and vascularization. The large number of variables inherently present in these interactions has historically been a primary challenge for experimental investigation.

Mathematical modeling provides a venue to systematically evaluate tumor and microenvironment interactions, including variation in ECM physical properties and macrophage polarization, to yield a system-level prediction of tumor behavior. Previous modeling has evaluated growth of liver metastases^10–12^, development of liver fibrosis^13,14^, and treatment of liver metastases via nanotherapy^15,16^. Recently, the kinetics of integrin receptor binding to hepatic ECM proteins were modeled^17^. However, the interaction of liver metastases, ECM and tumor-associated macrophages has not been extensively evaluated in a unified modeling framework. Here, we employ mathematical modeling to evaluate heterogeneous macrophage population interactions with tumor tissue^18^, considering ECM from normal and injured liver, and perform experiments with a mouse model to represent the *in vivo* human condition.

## Results

### Liver decellularization and cell migration

Tissues were evaluated both macroscopically and via histological analysis to confirm decellularization. Following 48 h decellularization, liver ‘ghosts’ were visually translucent and were sufficiently acellular via histologic assessment to continue with lyophilization step (S1 Figure).

**Figure 1:**
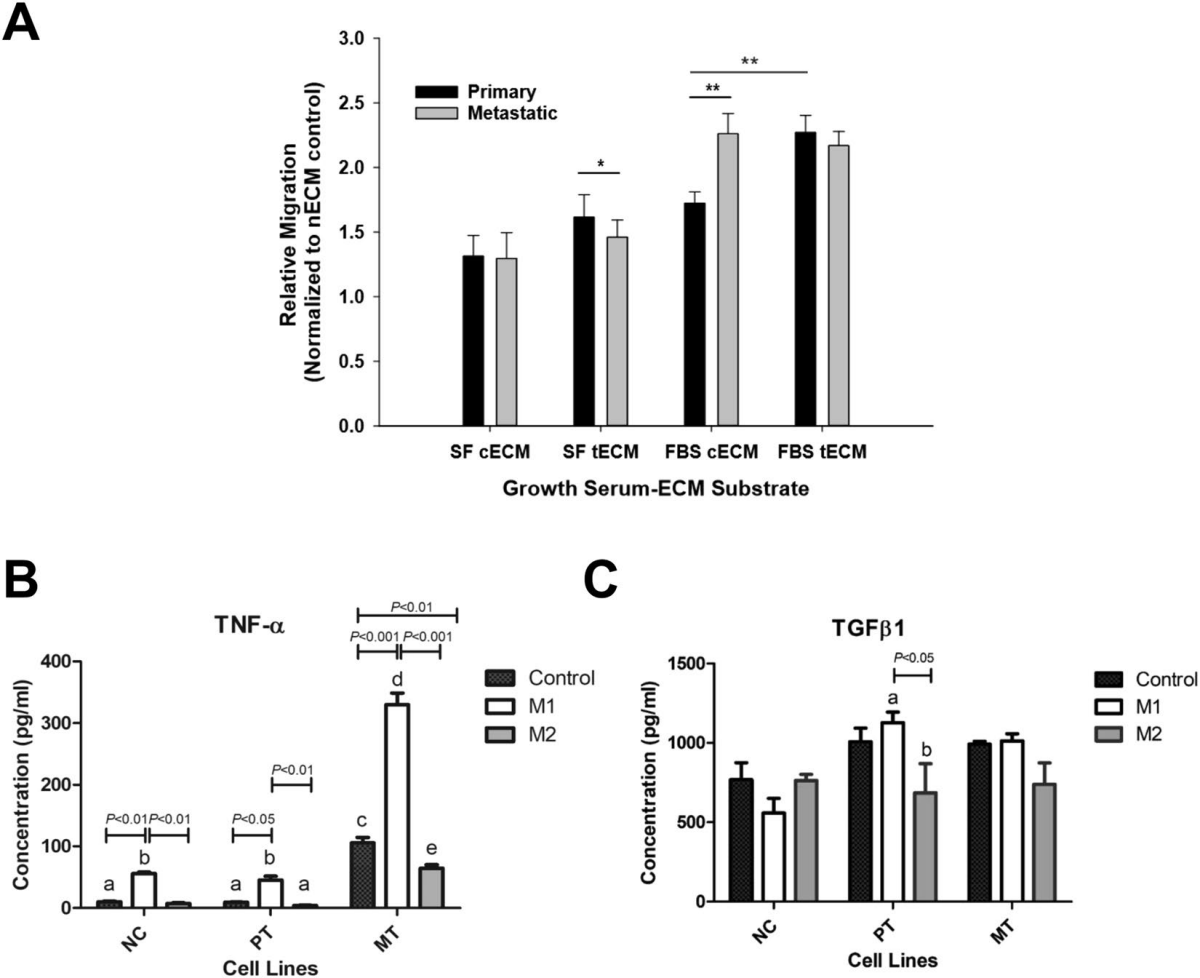
Transwell assays. (**A**) Relative migration of each tumor cell line on each ECM substrate, normalized to the respective “negative” control group of uncoated transwell membrane with no ECM (nECM) substrate coating. Five separate fields per slide were counted and averaged. Serum-free (SF) media controls were used to establish baseline migration with no chemoattractant in receiver well. Groups on the horizontal axis relate to growth conditions; within each group there is a representative bar for each cell line-ECM combination. Error bars represent the standard error of the mean, while horizontal bars highlight significant differences among pairwise comparisons. Primary tumor cells on transitional ECM (tECM) were included as a baseline for comparison, noting that they may not represent a biologically relevant case. Cells grown on FBS-supplemented control ECM substrate (“FBS cECM”) showed significant differences in relative migration between primary and metastatic cells (P < 0.001). Cells in serum-free tECM substrate (“SF tECM”) also showed significant differences in relative migration (P < 0.05). In supplemented media, relative migration of primary cells was shown to be different with respect to the ECM substrate (P < 0.001). (**B**) Macrophage-tumor cells indirect co-culture assay. TNF-α levels indicated a significant difference between the cases with M1-activated macrophages and the cases with naïve (“control”) or M2 macrophages (P ≤ 0.05). (**C**) Control transwells without tumor cells in the insert showed no differences in TGFβ1 levels across the treatment groups. NC: No tumor cells present; PT: with primary tumor cells; MT: with metastatic tumor cells.

Tumor cell migration data (normalized to control without ECM) are shown in Fig. 1A. Metastatic cell behavior was compared to that of primary cells. The statistical analysis shows that there were significant differences between the tumor cell type (P < 0.05), as well as between the growth serum-ECM substrate conditions (P < 0.001), and that the interaction between the two factors was significant (P < 0.001). With respect to growth serum-ECM substrate conditions, cells grown on serum-free transitional ECM substrate (“SF tECM”) showed significant differences between relative migration of primary and metastatic cells (P < 0.05). Cells in FBS-supplemented control ECM substrate (“FBS cECM”) also showed significant differences in relative migration (P < 0.001). In supplemented media, relative migration of primary cells was shown to be different with respect to the ECM substrate (P < 0.001).

### Simulation of TGFβ1 and TNF-α levels based on ELISA analysis

Indirect co-culture assay was used to evaluate the influence of tumor and macrophage cell populations on each other, with free exchange of cytokines across the transwell membrane, while restricting direct cell-cell contact between the two cultures. For all cases, including macrophage only, or with either primary or metastatic tumor cells, the TNF-α level was 5× higher with the M1 activated group than with the M2 group (Fig. 1B). The metastatic tumor cell co-culture exhibited the highest induction for all macrophage phenotypes. The level of TNF-α with M1 was ~10-fold higher than in the co-culture with the primary cell line, indicating that TNF-α production was enhanced with M1 in the presence of metastatic tumor cells. Accordingly, the production of TNF-α was set in the simulations for the metastatic tumors to be 10× higher than for the primary tumors, which used the baseline value in^18^. The levels of TGFβ1 in the different culture conditions were less dynamic, with only the co-culture with the primary tumor cell line exhibiting a significant difference between M1- and M2-activated macrophages (Fig. 1C), suggesting that TGFβ1 production increased slightly in the presence of M1. Consequently, in the simulations TGFβ1 production was maintained consistent between primary and metastatic tumors, on the same order of magnitude as the baseline value in^18^.

### Simulation of ECM production and vascularization

**Figure 2:**
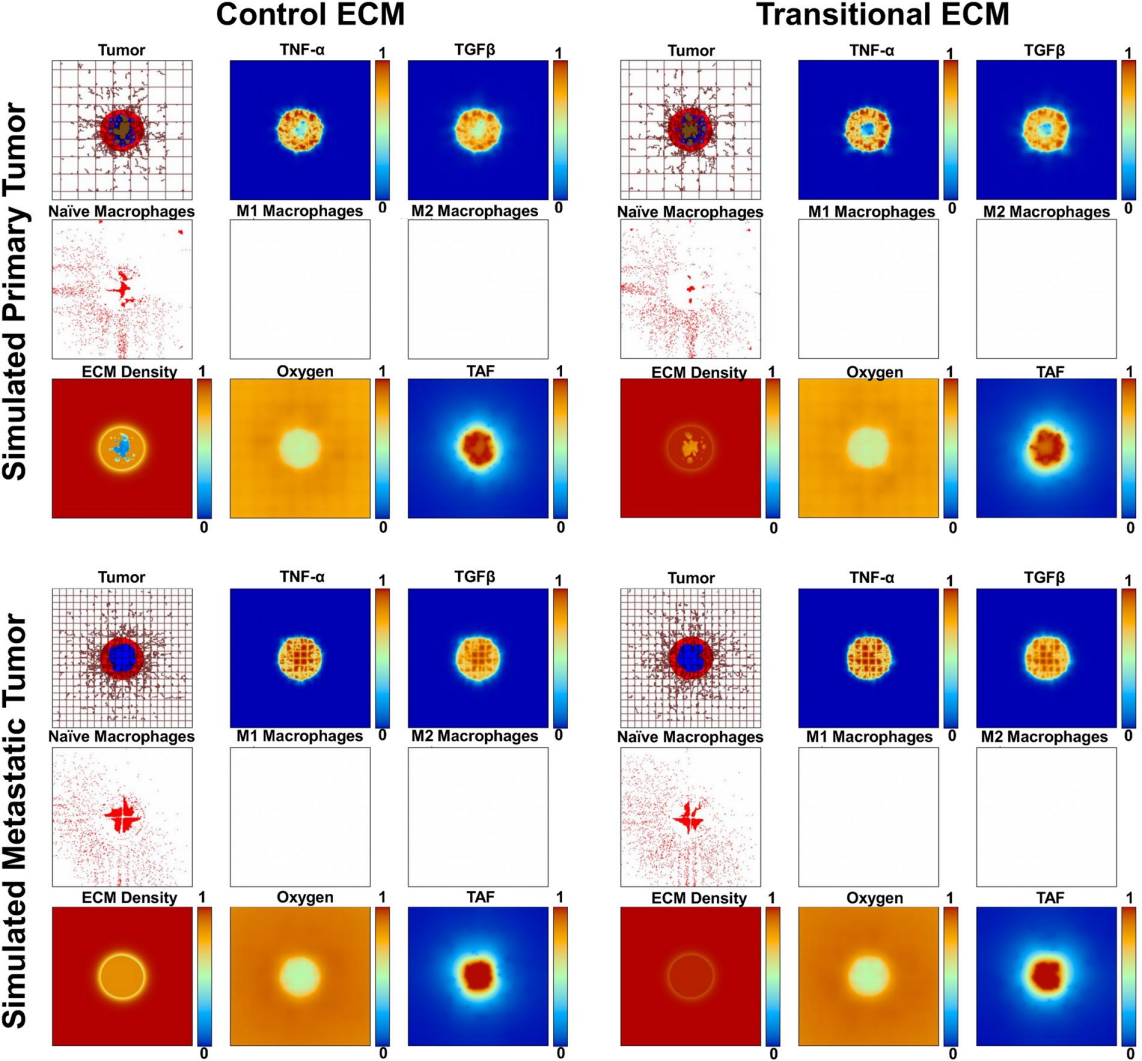
ECM-dependent simulations (naïve macrophage populations only). Simulated tumor growth at 13 d with ECM degradation/production ratio calibrated to simulate murine hepatic control ECM (cECM) and the transitional ECM (tECM) induced by alcohol exposure. The output matrix shows primary and metastatic tumors in the top and bottom rows, respectively; cECM and tECM simulations are shown in the first and second columns, respectively. Each 3 × 3 grid, comprised of nine 4 mm^2^ panels, shows the tumor and vessels (brown lines) in the top left corner, with proliferating regions in red, quiescent hypoxic regions in blue, and necrosis in brown. Both primary tumor simulations in the top row have larger areas of necrosis than the metastatic tumor simulations in the bottom row, which have mostly hypoxic cores. The vasculature grid for metastatic cells was calibrated to be denser, to recapitulate the high density of liver vasculature. In the middle and right corners of the top row, the simulated macrophage chemoattractants, TNF-α and TGFβ1, are shown in heat map as they are secreted in the tumor microenvironment. The middle leftmost panel of each grid shows the naïve macrophages extravasated from the surrounding vasculature. The middle center and rightmost panel of each grid show the density of Type 1 (M1) and Type 2 (M2) macrophages, respectively, polarized in the tumor microenvironment. In these simulations, the polarization is turned off in order to only evaluate the effect of the ECM variations. The bottom middle and leftmost panels of each grid show the simulated tumor oxygenation and tumor angiogenic factors (e.g., VEGF), respectively.

Data from transwell migration assay and hepatic matrisome^4^ were used to calibrate the ECM degradation/production ratio in the absence of polarized macrophages in the model. Accordingly, ECM production and degradation rates (S1 Table) were set so that ECM density for the tECM reflected the increased collagens and ECM proteins determined to comprise the transitional matrix. With only naïve (unpolarized) macrophages, this simulation assessed the effects of ECM modulations alone on tumor growth (Fig. 2). Primary tumors were also simulated with tECM as a baseline for comparison. For metastatic tumors, the vascular grid was set to a higher density than that for the primary tumors, to represent the highly vascularized hepatic environment. In addition, metastatic tumors were calibrated to have a lower necrotic threshold in order to yield a higher proportion of hypoxic tissue, as has been observed to occur for liver metastases^19^.

For primary tumors, there were regions of necrotic tissue, as can occur with lung cancer^20^, while metastatic tumors developed hypoxic cores by 13 d after tumor initiation, simulating hypoxic metastatic nodules^19^. Incorporating the calculated ECM production and degradation rates resulted in a higher ECM density for the tECM simulations relative to the cECM, recapitulating the transitional matrix determined via matrisome analysis for alcohol-exposed liver tissue^4^, and a higher ECM density for metastatic tumors compared to primary tumors. Macrophages clustered in higher numbers into the metastatic lesions, due to the higher hypoxia therein, and independent of the vascular grid density.

### Effect of macrophage differentiation on tumor growth

**Figure 3:**
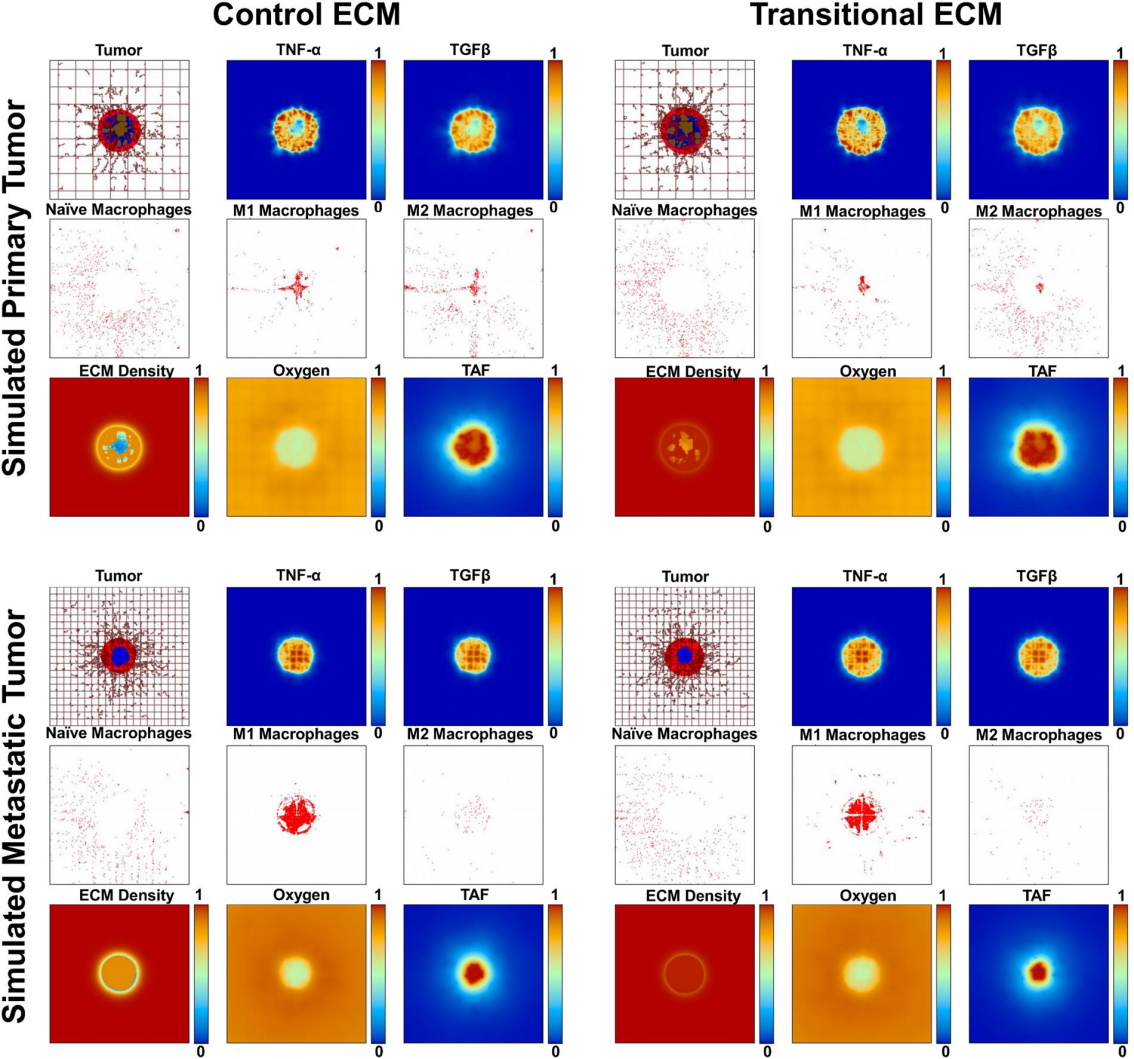
ECM-dependent simulations (with polarized macrophage populations). Simulated tumor growth at 13 d with macrophage polarization turned on (same grid panel descriptions as in Fig. 2). Both macrophage phenotypes appear to have penetrated the tumor mass. Metastatic tumors have a higher infiltration of M1 subtypes, which results in growth restriction relative to the primary cases.

Macrophage polarization was simulated to occur in the tumor microenvironment based on the levels of cytokines present therein, with TNF-α and TGFβ1 being representative molecules influencing M1^21^ and M2^22^ polarization, respectively (S2 Table). Levels of these simulated cytokines were determined from the ELISA analysis of the indirect co-culture. By 13 d after tumor initiation (Fig. 3), the primary tumor simulations showed a more even distribution of M1 and M2 subtypes, while metastatic tumors had a higher concentration of infiltrating M1 macrophages along the gradient of macrophage chemoattractants. The primary cECM simulation exhibited naïve macrophages distinctly clustered around the tumor mass. The M1 cytotoxic activity led to decreased tumor growth compared to the control simulations (Fig. 2) for both cECM and tECM metastatic lesions, with the latter regressing the most.

**Figure 4:**
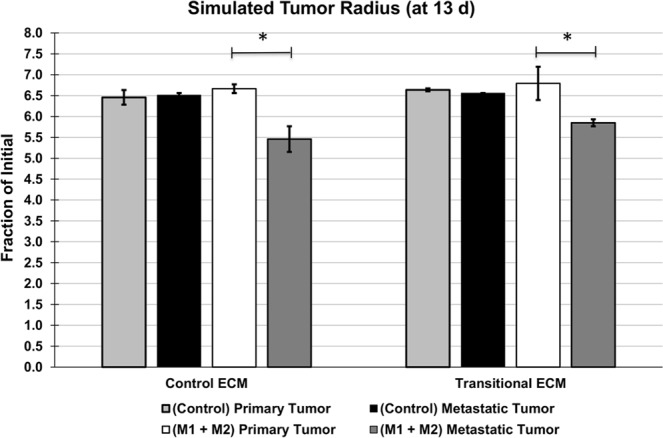
Simulated tumor size. Average tumor radius by 13 d. With polarized macrophages present, metastatic tumors had a smaller tumor radius than primary tumors. “Control” simulation tumor radii (with naïve macrophages only) were comparable for either ECM construct. Error bars represent standard deviation with n = 3; asterisk denotes significance calculated via student t-test, p-value < 0.05.

Simulated tumor growth under the various conditions was quantified in Fig. 4. Variation in the results was introduced by stochasticity in the vascular growth driven by angiogenesis as well as the macrophage polarization and movement, influenced by the concentrations of cytokines, as described in Methods. Whereas the “control” simulation groups (with naïve macrophages) for either ECM construct were comparable to each other, the polarized macrophage group for metastatic tumors was significantly smaller than for primary tumors in each ECM case by 13 d. In the simulations with polarized macrophages, metastatic tumor radius was 18% smaller than the primary tumor radius on cECM, while metastatic tumors were 14% smaller than primary tumors on tECM. There were no differences between primary or metastatic tumor cases across ECM constructs.

### Macrophage population dynamics

**Figure 5:**
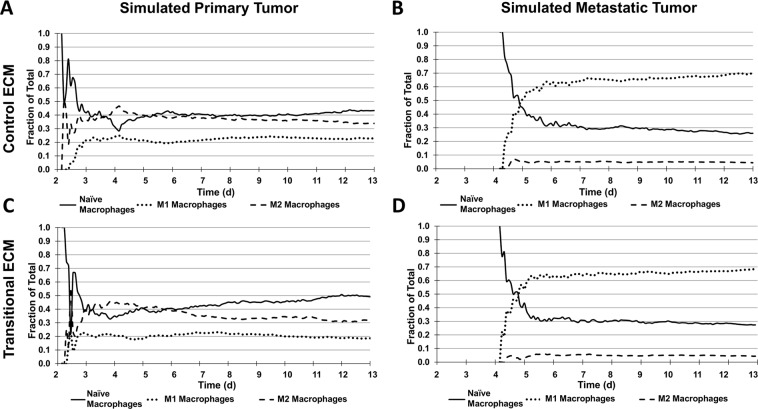
Simulated macrophage population fractions. Time evolution of macrophage subtype populations for (**A**) primary tumor on control ECM, (**B**) metastatic tumor on control ECM, (**C**) primary tumor on transitional ECM, and (**D**) metastatic tumor on transitional ECM. Primary tumor simulations of either ECM construct exhibited more balanced proportion of M1:M2 macrophage subtypes than metastatic tumors; on either ECM construct, M2 macrophages were more prominent than M1 for primary tumors. In contrast, M1 macrophages dominated for the metastatic tumors.

Primary tumor simulations run with either ECM construct exhibited a more balanced proportion of M1:M2 macrophages than metastatic tumors (Fig. 5). For cECM, M2 and M1 macrophages represented 34% and 23% of the total macrophage population by day 13 after tumor initiation, respectively, while for tECM, M2 macrophages were 32% and M1 were 19% of total macrophages. In contrast, M1 macrophages dominated for metastatic tumors, in response to the higher TNF-α production observed from the indirect co-culture assay. For cECM, M1 macrophages were 69% while M2 were 5% of total macrophages by day 13. For tECM, M1 macrophages represented 68% and M2 were 4% of the total population. These results indicate that with the parameter set evaluated in this study, the macrophage polarization was mainly influenced by the tumor type.

## Discussion

In addition to primary injuries (e.g., alcoholic liver disease), alcohol use also contributes to a broad range of secondary pathologies, including notably an increased risk of oncogenesis in several organs. Several studies having established links between alcohol consumption and cancers of the alimentary tract, as well as the breast, lung, and pancreas^23,24^. The fibrotic pathology associated with chronic ALD leads to enhanced inflammation and higher risk in certain cancers for increased aggressiveness^25–29^, and the associated induced dynamic tissue remodeling leads to desmoplasia and favorable conditions for tumor stromal overgrowth^30,31^. Increased risk of development of cancers has been determined via epidemiological studies and meta-analyses to be related to alcohol consumption^32,33^. The risk spectrum from light drinking to chronic alcohol consumption has been explored and a significant risk attributable to alcohol has been established for various primary cancers^34,35^.

In contrast to the known role of alcohol consumption in the development of primary tumors, whether or not alcohol exposure increases the risk to metastatic cancer distal from the primary site is unclear. Since cancer morbidity and mortality is largely attributable to metastases, rather than primary tumors per se, the mechanisms by which alcohol influences metastasis requires further investigation. Many primary cancers commonly metastasize to the liver at a higher proportion than almost all secondary sites save lymph nodes, including breast, colon/colorectal, lung, ovarian, and neuroendocrine tumors^36,37^. Hepatic metastases from colorectal cancer in particular are a significant clinical problem due to the frequency of synchronous lesions between the bowel and the liver^38,39^. Interestingly, alcohol consumption is a known risk factor for increasing metastasis to the liver, suggesting that early subclinical alcohol-mediated liver ECM remodeling may impact seeding and colonization of hepatic metastases^40^. In epidemiological studies, alcohol consumption has been identified as a significant independent risk factor for the development of colorectal liver metastases (CRLM)^41^. Retrospective analyses of clinical pathological reports have demonstrated a positive correlation between alcohol consumption and metastatic potential and patient outcomes^26,42^. However, whether this is a direct effect of alcohol consumption, or an indirect effect via increasing the risk of a primary tumor, is unclear. Furthermore, the specific mechanisms driving this correlation have yet to be elucidated and little is known about subclinical changes to microenvironment that influence organotropism of circulating tumor cells^43^.

Although the mechanisms of metastatic organotropism are poorly understood, it is clear that communication between cancer cells and the target microenvironment are critical. The interaction between tumor cells and macrophages was explored in the context of seeding to the selective “soils” of the homeostatic (control) lyophilized ECM vs. (transitional) lyophilized ECM derived tissue from liver experimentally injured by chronic alcohol exposure^4,43^. As a method of evaluating the impact of signaling on macrophage phenotype, the indirect transwell co-culture setup^44,45^, was employed using bone marrow-derived macrophages (BMDMs) seeded in various polarization states: naïve (MΦ) unpolarized, M1 polarized or M2 polarized. Migration assays on the two experimental ECM constructs showed that primary cells in supplemented media had increased relative migration (normalized to control without ECM) across membrane to lower chamber on lyophilized ECM derived from ethanol fed animals versus that from the control pair fed mice. We note that no residual cytokines and chemokines are expected in the respective ECM constructs following the decellularization approach and as assessed by proteomics analysis^4^, and thus the observed difference in migration would be due to the composition of the ECM. The simulations used the experimental data to modulate the ECM density, correlating to the increase in fibrous density that would occur in the remodeling of the ECM in response to alcohol exposure. Simulations were executed following calibration of production and degradation constants, resulting in a differential pattern of ECM, correlating to the alterations to ECM protein composition explored in^4^.

TAMs induce differential cytokine expression profiles in tumors, determined by population density and by location (i.e., vascularized versus hypoxic regions). Thus, simulating the proximity of TAMs to tumor stroma (classified by oxygenation threshold) and surrounding vasculature is important in determining their functionality^46,47^. The model of tumor lesions presented in this study provides visualization of positional macrophage clustering, and simulates tumor growth based on microenvironmental clues. The results illustrate the tumor makeup with regard to oxygenation and general nutrient availability, and define phenotypically the TAMs that share activation markers and tumor effector expression profiles with that of alternatively-activated M2 macrophages.

With levels of M1- and M2-associated cytokines calibrated to those found in the co-culture assay, simulations produced tumor growth results that illustrate the potent anti-tumoral potential of M1 macrophages in the system, as metastatic tumor growth for both control and transitional ECM was decreased compared to control simulations run with naive macrophages. This decreased growth resulted in part from higher simulated M1 numbers due to increased cytokine (TNF-α) levels, as experimentally measured in the cell culture supernatants. Both primary tumor simulations had larger necrosis than metastatic tumor simulations, which had mostly hypoxic cores. Accordingly, primary tumor tissue tended toward a balance between M1 and M2 populations, while metastatic tumors had a higher infiltration of M1 subtypes, which would result in growth restriction relative to all other cases.

Future work will consider a more detailed set of cytokines associated with macrophage-tumor interactions, in order to simulate more nuanced macrophage polarization and tumor effects. Additionally, simulating the macrophage polarization spectrum beyond a binary definition would more closely reflect the *in vivo* condition. Augmenting the model with data from further ECM experiments would help to more accurately calibrate to ECM changes that affect tumor cell attachment and subsequent immune activation. Parameters regarding stimulatory and inhibitory effects of ECM protein would add detail to the simulation of microenvironmental interactions. Here, simulated metastatic tumors included a higher vascularized environment to mimic the highly vascularized liver condition in addition to a lower necrotic threshold to simulate the more hypoxic nature of tumor liver metastases (S1 Table). The model coupling of the tumor and vascularization components then leads to different growth, angiogenic, and immune system dynamics between the primary and metastatic cases. Future work will consider more complex biological distinctions between these cases. Longer term, integration of patient tumor-specific ECM and immune cell data could provide the opportunity for *in silico* evaluation of therapeutic matrix perturbations with the goal to minimize liver tumor growth.

## Materials and Methods

### Animals

All mice were purchased from Jackson Laboratory (Bar Harbor, ME). Male C57BL6/J mice (6 wk) were utilized to harvest acellular liver scaffold following alcohol exposure (see below). Female B6129PF1/J mice (6 wk) were utilized for tumor allograft experiments. Mice were housed in a pathogen-free barrier facility accredited by the Association for Assessment and Accreditation of Laboratory Animal Care, and procedures were approved by the University of Louisville’s Institutional Animal Care and Use Committee. All experiments were performed in accordance with the relevant guidelines and regulations of the University of Louisville.

### Lieber-DeCarli alcohol diet maintenance

Following housing acclimation, mice were maintained on Lieber-DeCarli diet (Dyets, Inc., Bethlehem, PA) for 6w, with either ethanol-containing diet, isocaloric control diet containing maltose-dextrin; control mice were pair-fed to alcohol-fed mice to account for any consumption differences between the diets^48–51^. Both control and ethanol cohorts were acclimatized to the control diet for 2 d. The ethanol content in the ethanol-containing diet was subsequently increased in a step-wise fashion; 1%, and then 2% for 2 d each, 4%, and then 5% for 1w each, then finally 6% feeding for the final 3w. Animals were then sacrificed and liver tissue harvested for histological processing and ECM extraction.

### Histology

Liver tissues were either formalin fixed and embedded in paraffin (FFPE), or embedded frozen in optimal cutting temperature (OCT) prior to cutting at either 5 µm or 8 µm, respectively, and then mounted onto charged glass slides. FFPE sections were processed in Citrisolv (Thermo Fisher Scientific, Waltham, MA) and rehydrated via incubation in graded ethanol concentrations. Sections were stained with hematoxylin and eosin (H&E), Sirius red, or Mason’s Trichrome, before mounting with Permount (Thermo Fisher, Waltham, MA).

### Liver decellularization

Liver tissues were snap-frozen upon collection. To prepare for decellularization, 600 mg of frozen tissue was weighed and added to 45 ml sterile 1X PBS in 50 ml conical tubes. Tubes were put on shaker in cold room for gentle agitation overnight at 4 °C. Tissues were carefully removed from conical tubes with sterile forceps and transferred to new 50 ml conical tube containing 0.1% EDTA (ethylenediamine tetraacetic acid) in 10 mM Tris HCl at pH 8.0. Tubes were shaken at room temperature for one hour, then carefully removed with forceps and transferred to new 50 ml conical tube containing 0.1% SDS (sodium dodecyl sulfate) in 10 mM Tris HCl at pH 8.0. Tubes were shaken at room temperature for 24 h, then exchanged into fresh SDS buffer and shaken for another 24 h at RT.

After final 24 h of decellularization, tissues were washed three times by careful transfer to new 50 ml conical containing sterile 1XPBS and gentle agitation for an hour. Tissues were then transferred to 1.5 ml Eppendorf tubes, then centrifuged at 10,000 × g for 10 min. PBS was decanted and tissues were frozen overnight at −80 °C.

### Lyophilization

Tissues were kept on dry ice until lyophilization using a bench-top freeze dryer (SP Scientific, Warminster, PA) was initiated. Eppendorf tubes were opened and covered with parafilm, with small holes perforated using a small pipette tip. Tubes were placed inside lyophilization jar with cap open and parafilm opening exposed. Jar was attached to adaptor and secured. Lyophilization was initiated at −80 °C and 30 mTorr pressure, then evaporated for 48 h. Upon completion, the pressure was slowly released from jar prior to its detachment from apparatus. Tissue was hardened and white, and it was transferred to Eppendorf tube and ground with 10% pepsin in 0.1 M HCl. Sample was diluted to final concentration of 0.2 mg/ml in 0.2 M acetic acid.

### Culture plate coating

To coat cell culture plates, uncoated 12-well plates were treated with 0.2 mg/ml of lyophilized ECM in acetic acid, then incubated at 37 °C for one hour. Wells were then washed three times with sterile 1XPBS prior to seeding cells.

### BMDM cell culture

To harvest bone marrow-derived macrophages (BMDMs), mice were anesthetized with ketamine/xylazine (100/15 mg/kg i.p.) and sacrificed by exsanguination. Bone marrow cells were flushed from tibiae and femora of sacrificed mice using ice cold PBS, and then pooled from each cohort (4–6 mice) for propagation in cell culture using endotoxin-free RPMI 1640 medium supplemented with 10% fetal bovine serum (FBS; Gemini Bio-products, West Sacramento, CA) and 100 U/mL penicillin:100 µg/mL streptomycin (GE Health care, Wauwatosa, WI), with macrophage colony stimulating factor (M-CSF) supplementation via conditioned media from L-929 cells to induce differentiation into macrophages.

### Tumor cells and culture

Tumor cell lines designated 802T4 (derived from primary lung tumor in B6129S2/J mice) and 2691N1 (derived from lung to lymph node metastases in B6129S2/J mice presenting with primary lung tumors) were a kind gift from Dr. Winslow at MIT^52^. Cell lines were maintained with DMEM media with 10% FBS, and 100 U/mL penicillin:100 µg/mL streptomycin at 1%.

### Transwell migration assay

Serum-starved primary and metastatic tumor cell lines were seeded at concentration of 2 × 10^5^ on transwells with 8 µm porous membrane in chamber insert, coated with either control (cECM) or ethanol-fed transitional (tECM) lyophilized ECM liver tissue; uncoated wells were used as assay controls. Receiver plates contained media +/2% FBS serum as a chemoattractant. After 48 h, cells on lower surface of membrane were fixed with 4% paraformaldehyde, stained with 0.2% crystal violet in 100% ethanol, and counted manually, 10 fields per membrane, using automatic slide reader.

### Tumor-Macrophage indirect co-culture transwell assay

Transwell culture plates were utilized to create an indirect co-culture environment between tumor cell lines (either primary or metastatic) and BMDMs. A porous 0.4 µm transwell insert membrane was utilized to prevent migration of tumor cells from top chamber to bottom receiver well. Macrophages were first seeded to bottom receiver plate well at a density of 1 × 10^5^ cells/well and allowed to adhere overnight. Cells were then treated with either lipopolysaccharide (LPS) to induce M1-activation, or IL-4 for M2-activation; control cells were untreated naïve macrophages. After 24 h, tumor cells were seeded to the top chamber insert at a density of 5 × 10^4^ cells/insert; control inserts contained media only and no tumor cells. Following 48 h of indirect co-culture, cell supernatant was collected for further analysis.

### ELISA

Cell culture supernatant from indirect co-culture transwell assays was evaluated using Quantikine ELISA kits for TNF-α and TGFβ1 (R&D Systems, Minneapolis, MN) per manufacturer’s instructions. Assay controls, as well as cell culture media and experimental control samples were included for analysis.

### Statistical analysis

Comparative analysis of results from various experimental groups with their corresponding controls was performed using GraphPad Prism Version 5.03 for Windows (GraphPad Software, San Diego, CA). Transwell data were analyzed via 2-way ANOVA followed by Bonferroni post-hoc test to assess statistical significance between treatment groups, with tumor type and ECM substrate as the two independent variables. A *p* value < 0.05 was selected before the study as the level of significance.

The hypotheses tested were as follows:

- H10: The means of the tumor cell types are equal (null).
- H11: The means of the tumor cell types are different.
- H20: The mean cell counts of the ECM substrate type are equal (null).
- H21: The mean cell counts of the ECM substrate type are different.
- H30: There is no interaction between the tumor cell type and ECM substrate (null).
- H31: There is interaction between the tumor cell type and ECM substrate.

The cell counts for the ECM experimental groups (serum-free ECM and FBS-supplemented ECM) were each normalized by the mean of their respective control groups with uncoated transwells^53–55^, i.e. serum-free no-ECM and FBS-supplemented no-ECM, respectively.

### Simulations of tumor growth

The mathematical model is an application of the model presented in Mahlbacher *et al*.^18^, for which the interaction of macrophage polarization to M1- and M2-activated subtypes influence vascularized tumor progression.

#### Tumor growth component

The tumor growth component is based on^56^. The simulation space consists of a 2D evenly spaced grid of vasculature, representing a normal capillary network^57^. The microenvironment includes:

- Necrotic tissue: non-viable tumor oxygenation.
- Hypoxic tissue: non-proliferating tumor oxygenation.
- Normoxic tissue: sufficient oxygenation for tumor proliferation.
- Normal: non-cancerous tissue.

Tumor progression over time is modeled with proliferation dependent on changes in the microenvironment, including oncotic pressure, angiogenic factors, and oxygen concentration.

Tumor tissue advances (or regresses) with a certain velocity, *v*_*c*_, through the surrounding normal tissue and ECM, based on Darcy’s law^56^:

1 $${v}_{c}=-\,\mu \nabla P+{\chi }_{E}\nabla E,$$

where *µ* equates to tissue mobility (cell-cell, and cell-matrix linkages); *P* is oncotic pressure, *χ*_*E*_ is haptotaxis, and *E* is ECM density. If uniform tissue density is assumed, the growth velocity can be described via the net tumor proliferation rate *λ*_*p*_^56^:

2 $$\nabla \cdot {v}_{c}={\lambda }_{p}$$

The tumor main parameters are summarized in S1 Table.

#### Vasculature component

With nominal oxygen levels, proliferation distal from vasculature slows, hypoxic tissue regions are created once these levels reach the hypoxic tissue threshold (S1 Table), and from these regions are released a net balance of growth-promoting tumor angiogenic factors (TAF), which diffuse through tumor tissue into surrounding matrix, where they stimulate capillary sprouts from nearby vessels. Necrotic tissue is created once the oxygen levels drop below the necrotic threshold (S1 Table). The angiogenesis component^58^ includes tumor-induced neovascularization, flow through the vascular network, and mechanical and chemical effects of tumor growth on various network properties^56,57^.

#### Oxygen transport

Oxygen *σ* diffuses with coefficient *D*_*σ*_ from location of vessels, and is supplied at rates ${\lambda }_{neo}^{\sigma }$ and ${\lambda }_{pre}^{\sigma }$ from the neo- and pre-existing vasculature, respectively. Oxygen uptake by normal tissue has rate ${\lambda }_{{tissue}}^{\sigma }$, normoxic tumor tissue has rate ${\lambda }_{{tumor}}^{\sigma }$, while hypoxic tissue has rate *q*_*σ*_. Oxygen decays with rate ${\lambda }_{N}^{\sigma }$ in the necrotic region. Oxygen transport is approximated in quasi-steady state as^56^:

3 $$0=\nabla \cdot ({D}_{\sigma }\nabla \sigma )-{\lambda }^{\sigma }(\sigma )\sigma +{\lambda }_{ev}^{\sigma }({\bf{x}},t,{{\bf{1}}}_{vessel},p,\sigma ,h),\,{\rm{with}}$$

4 $${\lambda }^{\sigma }=\{\begin{array}{ll}{\lambda }_{tissue}^{\sigma }, & {\rm{outside}}\,{\rm{tumor}}\\ {\lambda }_{tumor}^{\sigma }, & {\rm{in}}\,{\rm{proliferating}}\,{\rm{region}}\\ {q}_{\sigma }(\sigma ), & {\rm{in}}\,{\rm{hypoxic}}\,{\rm{region}}\\ {\lambda }_{N}^{\sigma }, & {\rm{in}}\,{\rm{necrotic}}\,{\rm{region}}\end{array},$$

where **x** is position in space, *t* is time, **1**_*vessel*_ is the vessel characteristic function (equal to 1 at vessel locations, and 0 otherwise), *p* is the solid tumor pressure, and *h* is the hematocrit in the vascular network relating to extravasation of oxygen (following^56^). The extravasation function ${\lambda }_{ev}^{\sigma }$ is modulated by the extravascular interstitial pressure scaled by the effective pressure, as described in^59^.

Oxygen values are normalized with respect to the concentration in the vasculature, thus ranging from 0 to 1. Zero Neumann conditions are taken at the boundaries for all diffusion equations^56^.

#### Macrophages

Following^18^ and^15,16^, naïve macrophages were simulated to extravasate from the vasculature in proportion to the local concentration gradient of macrophage chemoattractants (e.g., TAFs produced by hypoxic tumor tissue^60^). Oxygen, pressure, and chemoattractant gradients direct macrophage migration through the interstitium. Macrophages are treated as discrete agents, simulated via a cellular automaton algorithm^18^.

Polarization to an M1 or M2 phenotype occurs in the tumor microenvironment, relative to cytokines in this microenvironment^18^ (S2 Table). Although in reality this polarization ranges over a spectrum of phenotypes^61^, with M2 populations capable of exhibiting a host of subtypes^62^, for simplicity the simulated macrophage phenotype is a binary state of either M1 or M2. Accordingly, the concentration of microenvironment cytokines, here represented by TNF-α and TGFβ1, is assumed to influence the polarization to M1^21^ and M2^22^ phenotypes, respectively. Under steady-state conditions, the overall mass balance for any cytokine concentration, *C* (dimensionless units), produced by viable (either proliferating or hypoxic) tissue is^63^:

5 $$0=\nabla \cdot ({D}_{C}\nabla C)+{\bar{\lambda }}_{production}^{\,C}\,(1-C){{\bf{1}}}_{\Omega }-{\bar{\lambda }}_{circulation}^{\,C}{{\bf{1}}}_{vessel}-{\bar{\lambda }}_{decay}^{\,C}\,C,$$

where *D*_*C*_ is diffusivity and ${\bar{\lambda }}_{production}^{\,C}$, ${\bar{\lambda }}_{circulation}^{\,C}$, and ${\bar{\lambda }}_{decay}^{\,C}$ are constant (non-dimensional) rates of cytokine production, circulation washout, and decay, respectively. Cytokine characteristics are summarized in S3 Table, based on prior work which classified protein diffusivity relative to molecular weight^63^.

#### Extracellular matrix

Tumor growth is modulated by ECM density *E*^57^:

6 $$\frac{\partial E}{\partial t}={\bar{\lambda }}_{production}^{\,E}\,\frac{1}{1+{k}_{p}E}{1}_{{\Omega }_{V}}+{\bar{\lambda }}_{sprout.production}^{\,E}\,\frac{1}{1+{k}_{p}E}{1}_{sprout.tips}-{\bar{\lambda }}_{{degradation}}^{\,E}\,\frac{EM}{1+{k}_{d}E},$$

where ${\bar{\lambda }}_{production}^{\,E}$ and ${\bar{\lambda }}_{sprout.production}^{\,E}$ are rates of production by proliferating tumor tissue and sprouting capillary vessels during angiogenesis, respectively, *k*_*p*_ and *k*_*d*_ are production and degradation scaling constants, respectively, ${1}_{{\Omega }_{V}}$ and **1**_*sprout.tips*_ are respectively the locations of viable tumor tissue and capillary vessel sprout tips, and ${\bar{\lambda }}_{{degradation}}^{E}$ is degradation rate. The rates are modulated by the existing ECM density. Degradation is further modulated by density *M* of matrix degrading enzymes (MDEs), released by proliferating tumor cells and vascular endothelial cells to remodel the ECM. MDE concentration is^57^:

7 $$\begin{array}{c}\frac{\partial M}{\partial t}=\nabla \cdot ({D}_{M}\nabla M)+{\bar{\lambda }}_{production}^{\,M}\,(1-M){{\bf{1}}}_{{\Omega }_{V}}+{\bar{\lambda }}_{sprout.production}^{\,M}\,(1-M){{\bf{1}}}_{sprout.tips}\\ \,\,\,-{\bar{\lambda }}_{{degradation}}^{\,M}\,\frac{EM}{1+{k}_{d}E}-{\bar{\lambda }}_{{decay}}^{\,M}M\end{array}$$

where *D*_*T*_ is diffusion coefficient, ${\bar{\lambda }}_{production}^{\,M}$ and ${\bar{\lambda }}_{sprout.production}^{\,M}$ are rates of production by viable tumor tissue and sprouting capillary vessels, respectively, ${\bar{\lambda }}_{{degradation}}^{\,E}$ is rate of degradation as they are uptaken by ECM, and ${\bar{\lambda }}_{{decay}}^{\,M}$ is decay rate.

#### Effect of macrophages

In the simulations, M1 macrophages were calibrated to recapitulate data indicating their deeper migration into tumor tissue than M2 subtypes^16^; this effect was modeled as a concentric field of unit value at the tumor center and a zero value at the tumor periphery. Thus, the model is biased to direct M1 movement based on its distance from the center of the tumor lesion.

Effects of the M1 and M2 macrophages were quantified via secretion of nitric oxide (NO) and tumor growth factors, respectively. These effects, ${\lambda }_{M1}$ and ${\lambda }_{M2}$, are included in the overall proliferation term^18^ with values depending on the respective tissue regions:

8 $${\lambda }_{p}=\{\begin{array}{l}non-tumor\,tissue:\,0\\ proliferating:({\lambda }_{M}+{\lambda }_{M2})\sigma -({\lambda }_{A}+{\lambda }_{M1})\\ hypoxic:{\lambda }_{M2}\sigma -({\lambda }_{A}+{\lambda }_{M1})\\ necrotic:-\,{G}_{N}\end{array},$$

where *λ*_*M*_ is tumor native mitosis rate, *σ* is local oxygen concentration calculated by Eq. 3, and *λ*_*A*_ is the native apoptosis rate. The non-dimensionalized cell degradation rate in the necrotic region is *G*_*N*_, assuming constant degradation of cellular debris and removal of associated fluid^57^.

The M1 anti-tumoral effect, *λ*_*M*1_ is simulated to affect tissue proportional to the release rate *λ*_*NO*_, of NO in the immediate vicinity of the macrophage (1_*M*1_), since NO has a short half-life *in vivo* with limited diffusion distance. M1 cytotoxicity is modeled to affect both proliferating (cycling) and hypoxic (quiescent) tissue, as this cell death is cell-cycle independent:

9 $${\lambda }_{M1}={\lambda }_{NO}{{\bf{1}}}_{{\boldsymbol{M}}{\bf{1}}}.$$

M2 growth factor positively affects the proliferating region as follows^18^:

10 $$\frac{d{\lambda }_{M2}}{dt}={\lambda }_{F}F(1-({\lambda }_{M}+{\lambda }_{M2})),$$

where *λ*_*M*2_ is the proliferation rate related to the concentration, *F*, of diffusible M2 growth factor. The effect of the M2 growth factor on the tumor proliferation is *λ*_*F*_.

M2 macrophages can also stimulate the proliferation of hypoxic tumor tissue, which is simulated to occur at lower rates than in proliferating tissue. The M2 macrophage-secreted tumor growth factor concentration, *F*, can transiently lower the local viable oxygen threshold as follows^18^:

11 $$\frac{d{Q}_{OL}}{dt}={\lambda }_{OL}\cdot (1-F)\cdot ({\bar{Q}}_{OL}-{Q}_{OL,current})-{\lambda }_{OT}\cdot F\cdot ({Q}_{OL,current}-{Q}_{OL,min}),$$

where *Q*_*OL*_ is the quiescence oxygen level, *λ*_*OL*_ is the quiescence oxygen level recovery rate back to the standard level, ${\bar{Q}}_{OL}$, ${Q}_{OL,current}$ is the current quiescence oxygen level, *F* is the local concentration of M2 growth factor ([0,1]; dimensionless units), *λ*_*OT*_ is the M2 growth factor effect rate on the lowering of the viable oxygen threshold, and ${Q}_{OLmin}$ is the lower bound of the quiescence oxygen level. Effective oxygen levels are set to ${\bar{Q}}_{OL}$ if they exceed ${\bar{Q}}_{OL}$, and to ${Q}_{OLmin}$ if less than ${Q}_{OLmin}$.

Macrophage-associated parameters are summarized in S4 Table, with values set as in Mahlbacher *et al*.^18^ or otherwise calibrated to correlate simulated tumor growth to experimental endpoints.

Following calibration of tumor, vascular, and macrophage cytokine parameters, the effects of ECM density and macrophage polarization on tumor growth were evaluated as in Table 1. Mirroring the experimental setup, primary tumors on tECM were included as a baseline for comparison, although they may not represent a biologically relevant case.

**Table 1:** Model conditions for simulation experiments.

Simulations		Case 1	Case 2	Case 3	Case 4
With naïve macrophages	ECM	cECM	tECM	cECM	tECM
	Tumor Type	Primary	Primary	Metastatic	Metastatic
With polarized macrophages	ECM	cECM	tECM	cECM	tECM
	Tumor Type	Primary	Primary	Metastatic	Metastatic

Simulation cases with naïve macrophages assess tumor response to differential ECM composition. Cases with polarized macrophages explore the response to heterogeneous macrophage populations. cECM: control (normal) ECM; tECM: transitional ECM.

## Supplementary information

Supplementary Information

## Data Availability

All data generated or analyzed during this study are included in the published article and its Supplementary Information files.
