# Supplementary material for: Computational/experimental evaluation of liver metastasis post hepatic injury: interactions with macrophages and transitional ECM
Source: Sci Rep. 2019 Oct 21;9:15077. doi: 10.1038/s41598-019-51249-y (PMC6803648; doi:10.1038/s41598-019-51249-y)
Supplement: Supplementary file 1 — Supplementary Information [file 41598_2019_51249_MOESM1_ESM.pdf]

Supporting Information for:

Computational/experimental evaluation of liver metastasis post hepatic injury: interactions with macrophages and transitional ECM

Shanice V. Hudson[1,2], Hunter A. Miller[2], Grace E. Mahlbacher[2], Douglas Saforo[1,4], Levi J. Beverly[1,2,4], Gavin E. Arteel[3,§], Hermann B. Frieboes[1,2,4,§]*

[1]Department of Pharmacology and Toxicology, University of Louisville, Louisville, KY 40202, USA

[2]Department of Bioengineering, University of Louisville, KY 40208, USA

[3]Department of Medicine, Division of Gastroenterology, Hepatology, and Nutrition, University of Pittsburgh, Pittsburgh, PA 15261, USA

[4]James Graham Brown Cancer Center, University of Louisville, KY 40202, USA

[§]Joint senior authorship

Differentiation and Movement of Macrophages

Following the description in [1], as naïve macrophages $M\phi$ extravasate from the vasculature into the tumor tissue, they come into contact with proteins in the tumor microenvironment that influence their differentiation. The respective differentiation rate R_i depends on the size of the interval that a randomly generated number may fall into; hence, the differentiation probabilities depend on the protein concentrations:

$$R_{M1} \propto k_{M1} \cdot C_{M1f}$$
$$R_{M2} \propto k_{M2} \cdot C_{M2f} \qquad \text{[Equation S1]}$$

where k_{M1}, k_{M2} are intensity coefficients tuned to reflect the relative prevalence of M1 or M2 differentiating monocytes infiltrating the tumor (**S4 Table**), C_{M1f} and C_{M2f} are the local concentrations of cytokines and other factors favorable to M1 or M2, differentiation, respectively, released by the viable (proliferating or hypoxic) tumor regions. Here, TNF-α and TGFβ1, are taken as representative tumor microenvironment molecules that influence polarization towards an M1 [2] and M2 [3] phenotype, respectively (**S2 Table**).

As described in [1], macrophages migrate through the tumor interstitium along gradients of oxygen, pressure, and chemoattractants. Movement can be in one of four directions in the 2D computational grid. The probability of movement in the x+1 direction is:

$$P_{x+1} = (M_O \cdot \Delta O_{x+1} + M_P \cdot \Delta P_{x+1} + M_C \cdot \Delta Chemo_{x+1}) \qquad \text{[Equation S2]}$$

where M_O, M_P and M_C are intensity coefficients representing the influence of oxygen concentration, pressure, and chemoattractant on macrophage movement (**S4 Table**), and $\Delta O_{x+1}, \Delta P_{x+1}$ and $\Delta Chemo_{x+1}$ are the difference in concentration of the factor of

interest from the current point to the direction in question. The same calculations are made for the remaining three directions in the 2D grid.

Supplementary Tables

S1 Table. Values for model main parameters (from [1]). All other tumor parameters are as in [4]. *Non-dimensionalized by O_2 diffusivity (1×10^{-5} $cm^2 s^{-1}$ [5]).

**Parameter**	**Value**	**Reference**
Hypoxic tissue threshold (primary tumors)	0.5750	[6]
Necrotic tissue threshold (primary tumors)	0.5700	[6]
Host vessel grid (primary tumors)	8x8	[6]
Hypoxic tissue threshold (metastatic tumors)	0.5750	[7]
Necrotic tissue threshold (metastatic tumors)	0.5325	[7]
Host vessel grid (metastatic tumors)	19x19	[7]
O_2 diffusivity	1*	[4]
O_2 vascular transfer rate	5*	[4]
Normoxic O_2 uptake rate	1.5*	[4]
Hypoxic O_2 uptake rate	1.3*	[4]
Microenvironment O_2 uptake rate	0.12*	[4]
O_2 decay rate	0.35*	[4]
cECM production constant	5	Calibrated from matrisome analysis [8]
cECM degradation constant	1	Calibrated from matrisome analysis [8]
tECM production constant	10	Calibrated from matrisome analysis [8]
tECM degradation constant	0.1	Calibrated from matrisome analysis [8]

S2 Table. Macrophage-associated cytokines simulated in the tumor microenvironment (adapted from [1]). *Diffusivity assumed similar to IL-10 in [1].

**Cytokine**	**Associated with:**	**Source**	**MW (kDa)**	**Diffusivity (as fraction of TAF diffusivity)**
TNF-α	M1 polarization	Hypoxic and proliferating tumor tissue	17	3.7606*
TGF-β	M2 polarization	Hypoxic and proliferating tumor tissue	13	3.7606*
NO	Local tumor necrosis	M1	0.03	0

S3 Table. Model rate parameters for macrophage-associated cytokines adapted from [1]. Washout and decay rates are generically applied to all cytokines, *C* (based on proteomic analysis in [9]). *Value non-dimensionalized by O_2 diffusivity (1 x 10^{-5} cm^2 s^{-1} [5]). **Value rescaled by the production rate of VEGF-A (VEGF-165) protein.

Parameter	Function	Value
$\bar{\lambda}^{C}_{circulation}$	Wash-out rate into vasculature	0.006*
$\bar{\lambda}^{C}_{decay}$	Decay rate	0.001*
$D_{TNF-\alpha}$	Diffusivity for TNF-α	0.005*
$D_{TGF-\beta}$	Diffusivity for TGF-β	0.005*
$\bar{\lambda}^{TNF-\alpha}_{production}$	Production rate of TNF-α (primary tumors)	1.0**
$\bar{\lambda}^{TNF-\alpha}_{production}$	Production rate of TNF-α (metastatic tumors)	10.0**
$\bar{\lambda}^{TGF-\beta}_{production}$	Production rate of TGF-β (primary tumors)	1.0**
$\bar{\lambda}^{TGF-\beta}_{production}$	Production rate of TGF-β (metastatic tumors)	1.0**

S4 Table. Characteristics of macrophage model parameters (adapted [1]). *Value non-dimensionalized by O_2 diffusivity (1 x 10^{-5} cm^2 s^{-1} [5]).

**Parameter**	**Description**	**Value**	**Reference**
Parameters Related to Tumor Growth			
λ_M	Tumor native proliferation rate (day^{-1})	0.5	[7]
λ_{OL}	Recovery rate of quiescent oxygen level	0.05*	[10]
λ_{OT}	M2 induced lowering viable O_2 threshold rate	200 /s	[10]
λ_{rec}	Recovery rate of λ_{M2} to zero	0.1*	[10]
λ_F	M2 induced proliferation rate	1000 /s	[10]
λ_{NO}	M1 nitric oxide induced death rate	3 /s	[11]
G_N	Cell degradation rate in necrotic region	0.3*	[7]
Differentiation Scaling Coefficients			
k_{M1}	M1 macrophage (primary tumors)	20	[10]
k_{M2}	M2 macrophage (primary tumors)	20	[10]
k_{M1}	M1 macrophage (metastatic tumors)	200	Scaled based on experimental data
k_{M2}	M2 macrophage (metastatic tumors)	20	28
Movement Scaling Coefficients			
M_O	Effect of oxygen on macrophage movement	1000	[7]
M_P	Effect of oxygen on macrophage movement	350	[7]
M_C	Chemotactic macrophage movement	500	[7]

Supplementary Figure

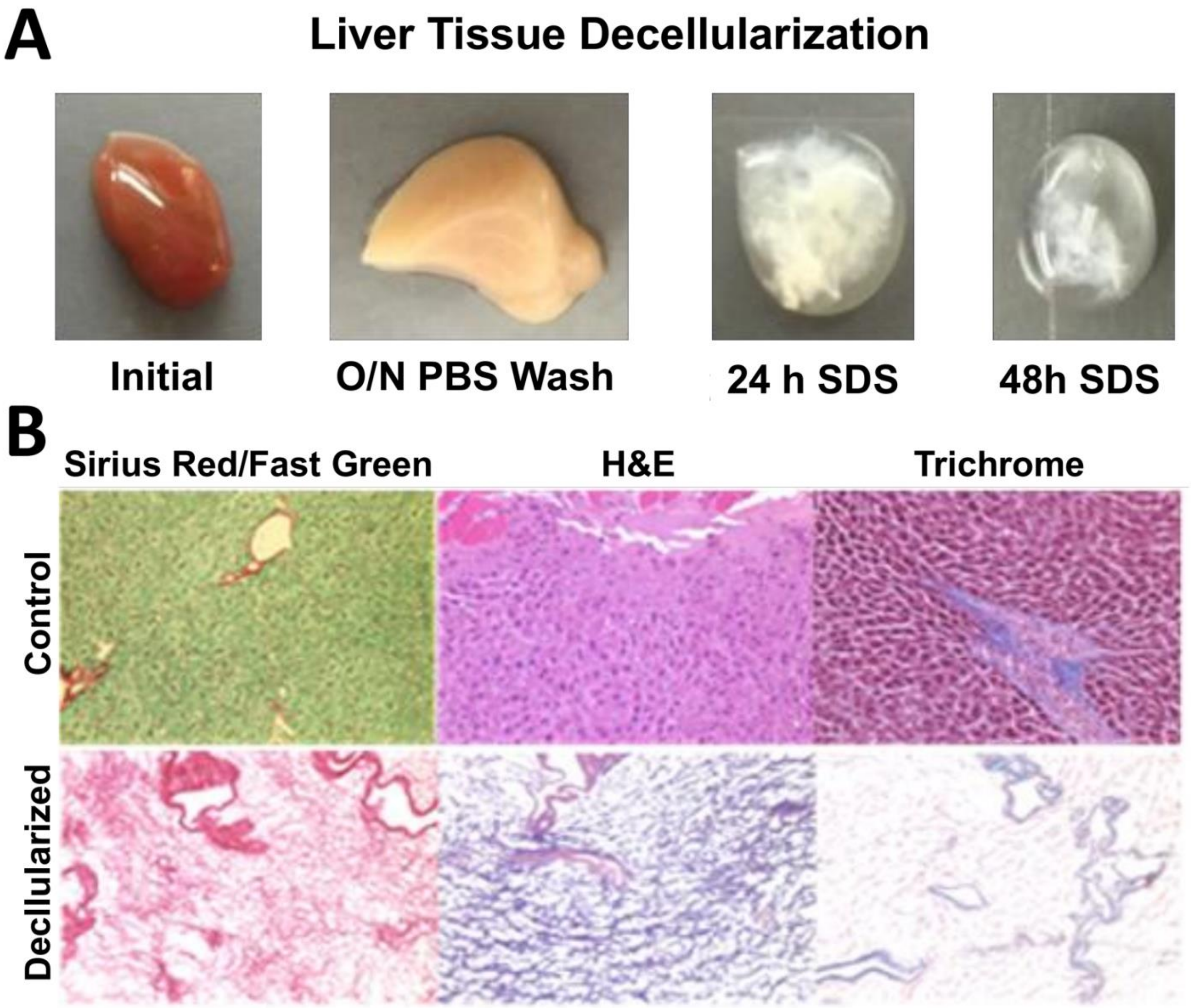

S1 Figure. Decellularization of liver tissue.

Gross pathology and histological analysis of decellularized liver tissue. (A) Tissues at each step of decellularization; at 48h, incubation in SDS solution yields translucent tissue “ghost” prepared for lyophilization. (B) Histological analysis of decellularized liver tissues. Top row shows control livers; bottom row represents decellularized samples. In the first column, Sirius Red/Fast Green collagen staining shows normal tissue in top row, with red staining of collagen, reticulin fibers, and basement membrane, and green

staining of non-collagenous proteins, while the decellularized sample in bottom row shows only collagenous material remaining. The second column shows H&E stain of normal tissue (top), and denucleation of acellular samples in the bottom row. The third column shows Trichrome stain of normal tissue (top), with nuclei stained in black, cytoplasm stained red, and collagen in blue; the acellular sample (bottom) shows removal of nuclei and cytoplasm, with retention of collagen in blue.

Reference List

1 Mahlbacher, G., Curtis, L. T., Lowengrub, J. & Frieboes, H. B. Mathematical modeling of tumor-associated macrophage interactions with the cancer microenvironment. *Journal for immunotherapy of cancer* **6**, 10, doi:10.1186/s40425-017-0313-7 (2018).

2 Ruytinx, P., Proost, P., Van Damme, J. & Struyf, S. Chemokine-Induced Macrophage Polarization in Inflammatory Conditions. *Frontiers in immunology* **9**, 1930, doi:10.3389/fimmu.2018.01930 (2018).

3 Zhang, F. *et al.* TGF-beta induces M2-like macrophage polarization via SNAIL-mediated suppression of a pro-inflammatory phenotype. *Oncotarget* **7**, 52294-52306, doi:10.18632/oncotarget.10561 (2016).

4 Wu, M. *et al.* The effect of interstitial pressure on tumor growth: coupling with the blood and lymphatic vascular systems. *Journal of theoretical biology* **320**, 131-151, doi:10.1016/j.jtbi.2012.11.031 (2013).

5 Nugent, L. J. & Jain, R. K. Extravascular diffusion in normal and neoplastic tissues. *Cancer research* **44**, 238-244 (1984).

6 van de Ven, A. L. *et al.* Integrated intravital microscopy and mathematical modeling to optimize nanotherapeutics delivery to tumors. *AIP advances* **2**, 11208, doi:10.1063/1.3699060 (2012).

7 Leonard, F. *et al.* Enhanced performance of macrophage-encapsulated nanoparticle albumin-bound-paclitaxel in hypo-perfused cancer lesions. *Nanoscale* **8**, 12544-12552, doi:10.1039/c5nr07796f (2016).

8 Massey, V. L. *et al.* The hepatic "matrisome" responds dynamically to injury: Characterization of transitional changes to the extracellular matrix in mice. *Hepatology (Baltimore, Md.)* **65**, 969-982, doi:10.1002/hep.28918 (2017).

9 Frieboes, H. B., Curtis, L. T., Wu, M., Kani, K. & Mallick, P. Simulation of the Protein-Shedding Kinetics of a Fully Vascularized Tumor. *Cancer informatics* **14**, 163-175, doi:10.4137/cin.S35374 (2015).

10 Leonard, F. *et al.* Macrophage Polarization Contributes to the Anti-Tumoral Efficacy of Mesoporous Nanovectors Loaded with Albumin-Bound Paclitaxel. *Frontiers in immunology* **8**, 693, doi:10.3389/fimmu.2017.00693 (2017).

11 Yuan, A. *et al.* Opposite Effects of M1 and M2 Macrophage Subtypes on Lung Cancer Progression. *Scientific reports* **5**, 14273, doi:10.1038/srep14273 (2015).
